# Aspergillosis: An Unwanted Tenant of Lung Cavity in an Immunocompromised Host

**DOI:** 10.7759/cureus.23708

**Published:** 2022-03-31

**Authors:** Anuradha Sakhuja, Dhan B Shrestha, Anurag Adhikari, Wasey Ali Yadullahi Mir, Mtanis Khoury, Shan-Ching Ying, Mohammed Kassem

**Affiliations:** 1 Department of Internal Medicine, Mount Sinai Hospital, Chicago, USA; 2 Intensive Care Unit, Nepal Korea Friendship Municipality Hospital, Madhyapur Thimi, NPL; 3 Department of Internal Medicine, Mount Sinai Medical Center, Chicago, USA; 4 Department of Pathology, Mount Sinai Hospital, Chicago, USA; 5 Department of Hematology and Oncology, Mount Sinai Hospital, Chicago, USA

**Keywords:** aspergillus, lungs abscess, immunocompromised, aspergilloma, breast carcinoma

## Abstract

Immunocompromised status predisposes an individual to infection from bacteria, fungi, and viruses that are otherwise uncommon. The presence of carcinoma and the use of chemotherapy weakens one’s immune system and leads to opportunistic infections of many kinds. Aspergilloma is a fungal ball that grows inside a primary cavitary lesion within the pulmonary parenchyma. Generally, immunocompromised individuals have severe and invasive infections from *Aspergillus. *Here, we present a case report of a female with breast carcinoma undergoing chemotherapy who previously had a lung abscess with *Klebsiella. *During her subsequent presentation, she was detected to have aspergilloma along with multi-drug-resistant organisms in the lung abscess along with metastasis of breast carcinoma and lung squamous cell carcinoma encapsulating the fungal ball.

## Introduction

Aspergillus is a fungus whose mycelium is usually found in the soil [1]. The conidia from the fungus are spread into the air from where they are inhaled into the lungs [2]. A healthy individual usually does not get infected. *Aspergillus fumigatus* and *Aspergillus niger* are the two common species causing illness in humans [3]. Depending upon the host's immune status, a wide spectrum of diseases may be manifested in humans [4]. Immunocompetent hosts generally are either asymptomatic or have allergic manifestations of infection. However, immunocompromised individuals have invasive diseases and are at risk of severe infections [4].

## Case presentation

A 50-year-old African American female with a past medical history of stage III A metastatic right-sided breast cancer, estrogen receptor/progesterone receptor (ER/PR) positive, human epidermal growth factor receptor 2 (HER-2) negative, diagnosed in 2016, presented to our emergency department in January 2021 with the complaint of shortness of breath and chronic productive cough with blood-tinged sputum ongoing for six months. She had a history of right lung upper lobe *Klebsiella* abscess in 2018 (which was treated with pigtail drain by interventional radiology, and an empiric antibiotic, later escalated based on sensitivity pattern). She was receiving chemotherapy for ER/PR positive stage IIIA breast cancer. She initially received doxorubicin/cyclophosphamide (four cycles) with taxol. However, chemotherapy was complicated by neuropathy and cytopenias. The patient underwent right mastectomy with axillary lymph node dissection and adjuvant radiation therapy due to the progression of the disease. Meanwhile, she received a combination of ribociclib, letrozole, and goserelin from March 2018 to March 2020, which was switched to carboplatin/gemcitabine (seventh cycle in December 2020).

**Figure 1:**
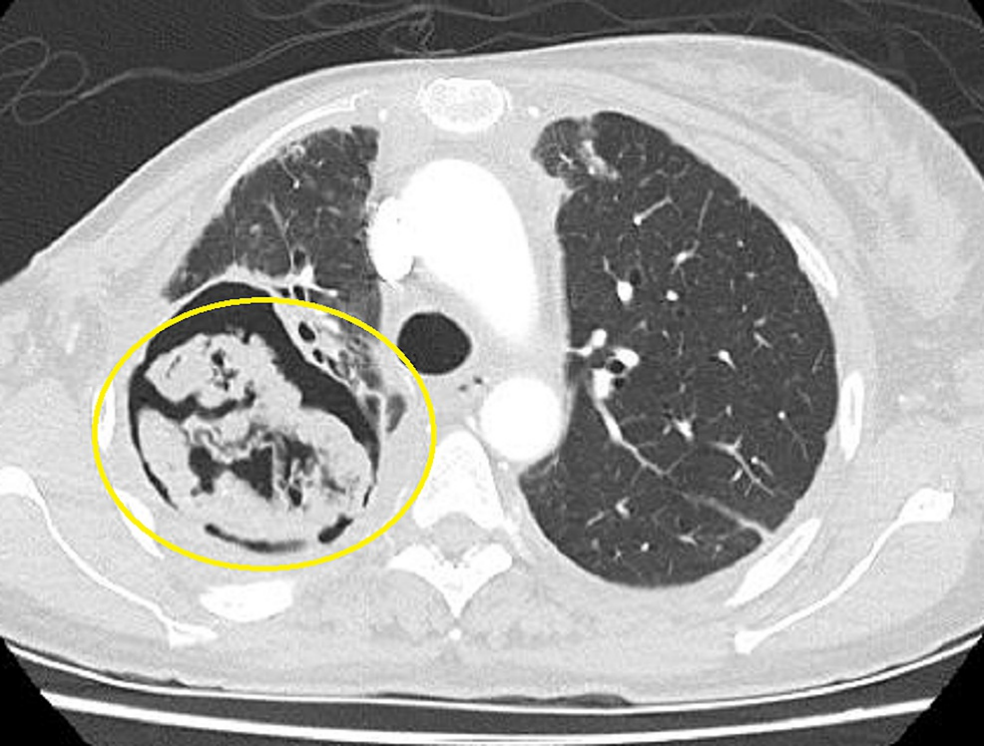
Transverse section of CT scan of the chest showing cavitary lesion on the right upper lobe

**Figure 2:**
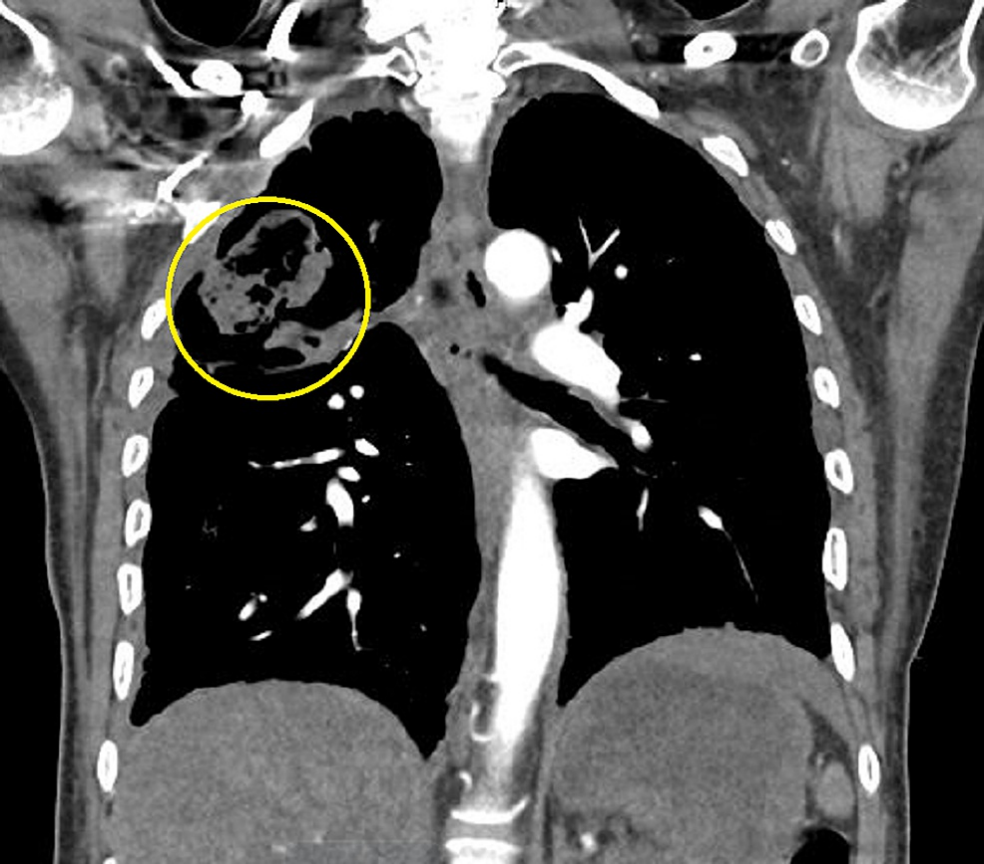
Coronal section of CT scan of the chest showing cavitary lesion in the right upper lobe of chest

**Figure 3:**
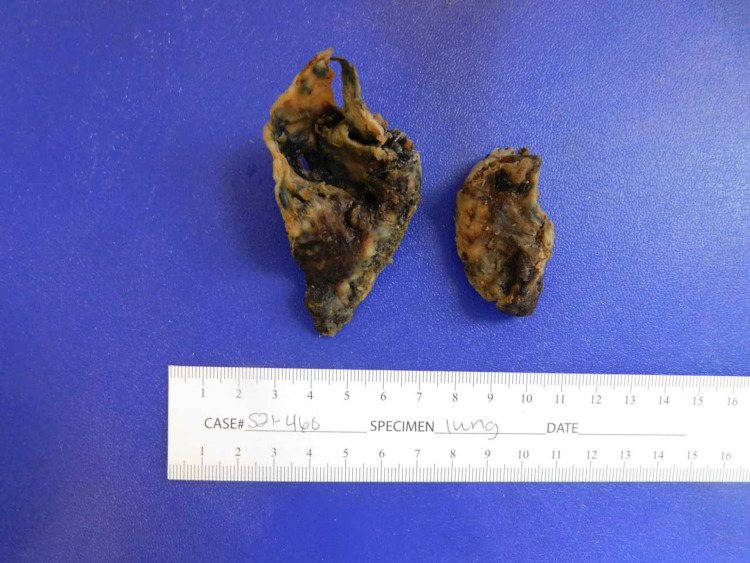
Part of the right lung forming right upper lobe abscess cavity

**Figure 4:**
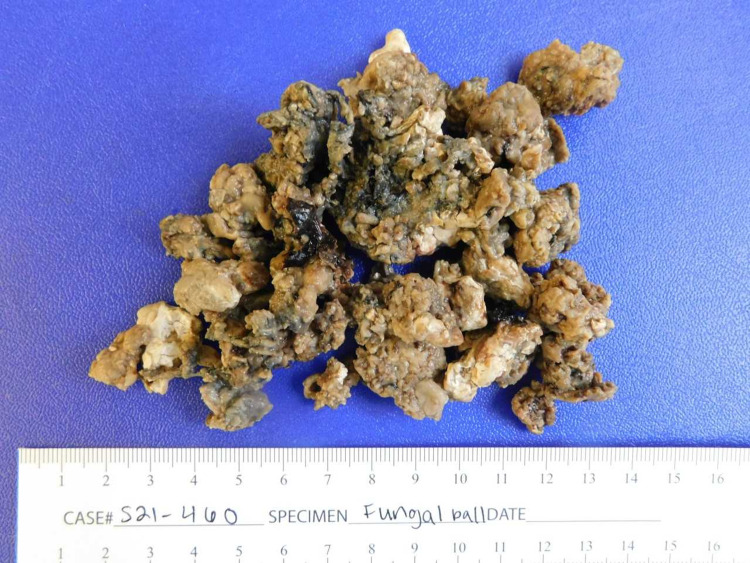
Gross tissue specimen forming fungal ball

**Figure 5:**
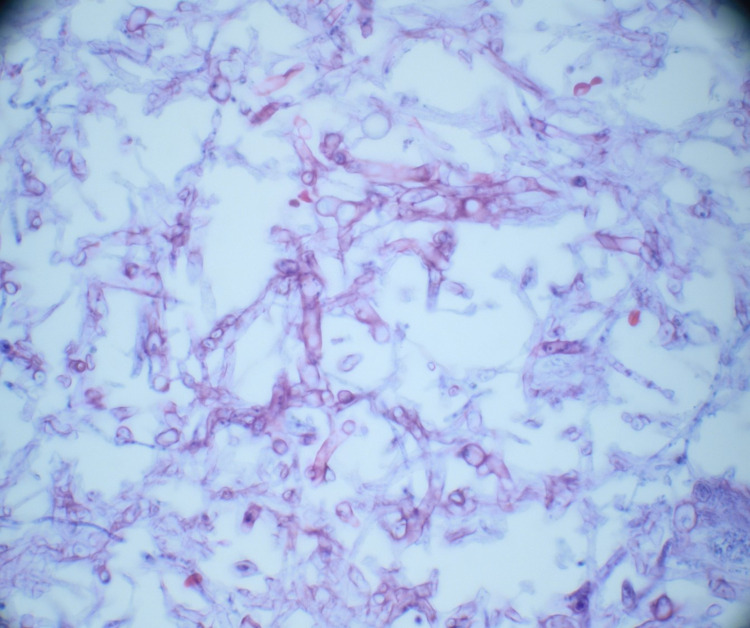
High power microscopic image showing fragments of septate fungal hyphae consistent with Aspergillus species in H&E stain H&E: hematoxylin and eosin

**Figure 6:**
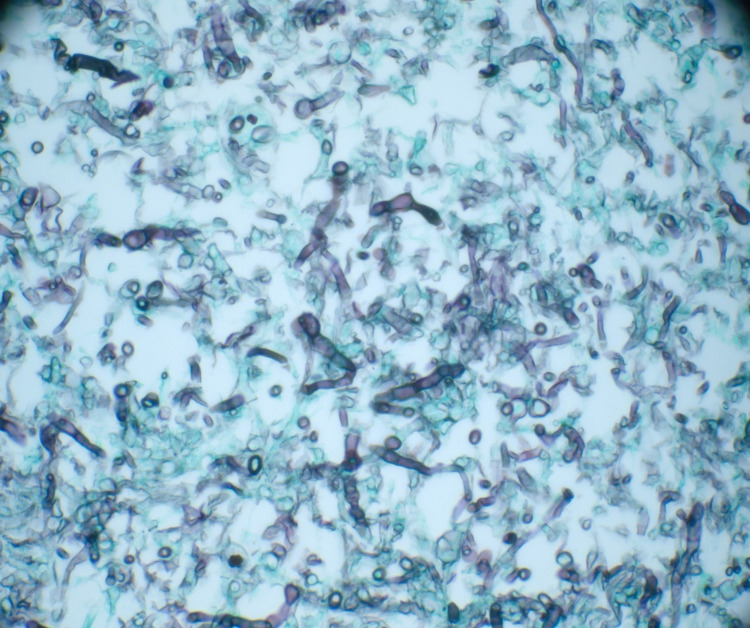
High power microscopic image showing fragments of septate fungal hyphae consistent with Aspergillus species in Giemsa stain

**Figure 7:**
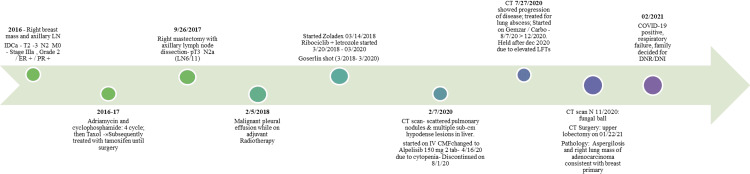
Timeline of the events

The computed tomography (CT) scan of the chest demonstrated a cavitary mass in the posterior segment of the right upper lobe measuring 7.4 x 6.9 x 7.9 cm with a nodular soft tissue (fungal ball) within the cavity measuring approximately 6.1 x 5.4 x 5.2 cm. It was highly suspicious for aspergilloma (Figure 1 and Figure 2). Bronchoscopy with bronchioalveolar lavage was done for persistent hemoptysis, which showed atypical squamous cells. Therefore, a parietal pleurectomy and wide local excision of the large right upper lobe abscess cavity were performed (Figure 3). Pathology of right upper lobe lung tissue was significant for ER/PR positive breast adenocarcinoma encapsulating fungal ball (Figure 4), demonstrating fragments of septate fungal hyphae consistent with *Aspergillus* species (Figure 5 and Figure 6) and gram-negative bacteria, *Pseudomonas fluorescens*, *Aspergillus flavus*, and *Prevotella oris*. The patient was responding well to the chemotherapy regimen and cefepime and voriconazole. Unfortunately, the patient succumbed to coronavirus disease 2019 (COVID-19) infection complications. The overall timeline of the case has been summarised in Figure 7. 

## Discussion

Lungs abscess is caused by liquefactive necrosis of the pulmonary parenchyma. The most common cause of lung abscesses is alcoholism [5]. Most lungs abscesses are polymicrobial, as seen in our patient. The noninvasive disease spectrum caused by *Aspergillus* is classified as chronic pulmonary aspergillosis (CPA) [6]. The pathogenesis of CPA involves a prior defect in mucociliary clearance due to structural lung disease with previous infections like tuberculosis implicated as a causative factor [4]. Aspergilloma is a type of CPA in which *Aspergillus* colonizes the cavity in the lungs. It consists of dead and living mycelium combined with inflammatory cells, components of degenerating epithelium, etc. [4].

The typical presentation of aspergilloma is hemoptysis, which can be self-limited or even massive [7,8]. Our patient, too, presented with hemoptysis, which was initially attributed to the use of apixaban. The diagnosis of aspergillosis is based on the presentation, radiographic features, and IgG antibodies to *Aspergillus* [9]. As seen in radiological evidence, aspergilloma is usually located in the upper lung fields as a solid round mass within a cavity [4]. Our patient had a fungal ball in the proximity of the bronchial artery, which was causing the hemoptysis.

However, the serum IgG antibodies to Aspergillus may be falsely negative in patients receiving corticosteroids or with infection by other species than *Aspergillus fumigatus* [3].

Our patient underwent surgical intervention due to co-existing hemoptysis. Even though the surgical resection of aspergilloma is associated with significant mortality and morbidity, our patient tolerated the procedure well [10,11]. Unfortunately, the outcome of coexisting COVID-19 infection and aspergillosis is poor [12]. Aspergilloma in solid cancer of lung and aspergilloma mimicking lung cancers are reported in the literature; however, aspergilloma in metastatic breast cancer to the lung as in our case has not been reported earlier to the best of our knowledge.

## Conclusions

Although immunosuppression due to malignancy and chemotherapy predispose to severe and invasive infection with *Aspergillus*, prior lung infection may also result in aspergillosis. The lung abscess associated with the fungal ball is generally polymicrobial. Co-infection with *Aspergillus* and COVID-19 turned out to be fatal for our patient.
